# Healthcare Profession Students’ Motivations for Learning About Community Organizing: A Thematic Analysis

**DOI:** 10.7759/cureus.46881

**Published:** 2023-10-12

**Authors:** Ryuichi Ohta, Akiko Yata, Chiaki Sano

**Affiliations:** 1 Community Care, Unnan City Hospital, Unnan, JPN; 2 Family Medicine, Community Nurse Company, Izumo, JPN; 3 Community Medicine Management, Shimane University Faculty of Medicine, Izumo, JPN

**Keywords:** curriculum reform, social determinants of health, preventive medicine, holistic health, student motivation, community engagement, healthcare professionalism, experiential learning, medical education, community organizing

## Abstract

Background

Medical care now emphasizes community health, prevention, health promotion, and collaboration. Integrating medical students into community health initiatives enhances their community health and student skills. In an aging multicultural population, the involvement of healthcare professionals in community health management is vital. However, medical education in Japan lacks sufficient exposure to community health issues. A program in Shimane Prefecture aimed to address this gap by revolutionizing medical education through community organizations.

Methods

This study employed a reflexive thematic analysis based on relativist ontology and constructivist epistemology. Participants aspiring to be healthcare professionals from Japanese high schools and universities were recruited from rural Shimane Prefecture. Computer-based questionnaires were used to collect data on participants’ reasons, motivations, and visions for community-organizing education. The thematic analysis followed Braun and Clarke’s approach and involved systematic coding, theme identification, and refinement.

Results

Three themes emerged from the analysis. In expanding hopes for unknown potential, participants sought improved communication skills, real-world understandings, and fresh perspectives and aimed to promote personal growth through community engagement. In acquiring activeness and new perspectives through connections with peers, hands-on learning and collaboration with peers with shared interests were motivating factors. Participants sought to generate inquiries and discover their activities. Regarding the desire to connect with diverse individuals driven by a strong curiosity about the community, participants aimed to learn community engagement techniques, understand community involvement methods, and explore the relationship between social issues and health.

Conclusion

Community-organizing education plays a pivotal role in shaping future healthcare professionals. Our analysis underscores the need for curriculum reform, including experiential learning and peer interaction, to facilitate a comprehensive understanding of health and community dynamics. Future studies should assess the long-term impacts of these experiences on students' careers and community health to contribute to advancements in medical education and community-oriented healthcare professionalism.

## Introduction

In recent years, the landscape of medical care has expanded beyond hospitals and clinics to include the entire community [1]. This shift can be attributed to the growing importance of promoting local residents’ health and preventive medicine and the need for strengthened collaboration in community health care [2]. Within the framework of community organization, it is believed that medical students’ active participation in community health initiatives can not only improve the health status of the community but also enhance their skills and broaden their perspectives [3]. In future aging and multicultural societies, it will be crucial for healthcare professionals to be involved in everyday health management in both medical institutions and the community [4].

Currently, opportunities for Japanese medical students to learn about health maintenance in a community context are limited [5]. Most healthcare professionals learn in medical institutions, with few opportunities to understand the relationship between community living and health [6]. To adapt to societal changes, it is necessary to provide opportunities for medical students to immerse themselves in community life and learn about health activities and behaviors therein [7]. Thus, educational content that allows students to learn alongside community residents is required.

Currently, a program offering medical students opportunities to participate in activities supporting community health is set to be launched in remote parts of Japan. In Shimane Prefecture, which is one of the most aged prefectures in Japan, a program targeting medical students in community organizations that facilitates resident activities and community health improvements is being initiated. In contrast to traditional educational curriculum about health care, this initiative can potentially enhance medical students' learning regarding health activities outside medical facilities and possibly create a new learning approach for medical schools.

However, this approach is a novel learning method for Japanese medical students, and their motivations and participation objectives may vary. Thus, developing a learning curriculum for community activities is crucial based on students’ objectives and motivations [8]. Understanding these factors could influence future emphases in medical education and professionalism. Moreover, this study provides new guidelines and methodologies for promoting medical students' education, training, and community participation. Therefore, this study aimed to elucidate the thoughts and motivations of professional healthcare students participating in community-organizing education.

## Materials and methods

This study utilized reflexive thematic analysis based on ontology and epistemology for research purposes. Professional healthcare students have diverse backgrounds and prior experiences [9]. They participate in community-organizing workshops from different perspectives, which can be contextual and self-directed. We examined relativist ontology and constructivist epistemology to investigate their perceptions of and motivations for learning concerning context. Thus, the thematic analysis was based on a qualitative framework [10].

Setting and participants

This study was conducted in Shimane, a rural prefecture in Japan comprising eight cities, five counties, 10 towns, and one village. The population of Shimane is 665,702, and the proportion of the population aged ≥65 years is 34.7% [11]. Social media and snowball sampling recruited participants from Japanese high schools and universities. The information of the education spread through the relationships among interested learners. In this study, participants consisted of 30 students from any grade or facility in Japanese universities and high schools who were motivated to become healthcare professionals and applied for community organizing and care education provided by the Community Nurse Company. There were 10 male and 20 female participants, and seven were from other Japanese prefectures. All participants were informed about the study's aim and agreed to participate after providing informed consent.

Planned community organizing and care education provided by a community nurse company

This study provided community nursing education. Community nursing does not simply provide medical care but weaves itself into the intricate tapestry of individuals' daily lives, fostering moments of happiness and contentment, a community-organizing method [12]. Individuals’ roles surpass their clinical duties as they delve into social, emotional, and spiritual support. The objectives of this study are to understand the distinctive perspectives and approaches in community nursing, advance a community organization plan by initiating first contact with residents, and strengthen axes of interest and passion for engaging with fellow residents in the community. Participants considered their plans for community organization by learning about community nursing. They met each other monthly and discussed their plans with other participants. They also communicated with various citizens and professionals who acted locally for community organizations to deepen their understanding of community organizations and to improve their community plans.

Data collection

Data were collected using computer-based questionnaires on sex, affiliation, reasoning, motivation for participation, and vision after learning. Regarding reasoning, motivation for participation, and vision after learning from education, the following questions were used in a free-writing format to investigate students’ perceptions and thoughts regarding community organizing learning: (1) “Why did you participate in this educational program?” (2) “How did you participate in this educational program?” (3) “How will you apply the learning and experience in this educational program to your future?” Each free-writing answer contained approximately 300 words. Data were collected between July and August 2023.

Analysis

Thematic analysis was selected as the data analysis method for this qualitative study. Braun and Clarke’s (2006) six-step approach to thematic analysis, which offers a systematic framework for identifying, analyzing, and interpreting patterns of meaning within the data, was adopted [10]. In the initial stage of familiarization with the data, two researchers (RO and AY) read the transcripts of the computer-based questionnaire sheets multiple times and noted their initial ideas. RO and AY conducted systematic coding across the entire dataset to generate the initial codes and generate relevant codes for the data about the research questions. Through discussions between RO and AY, codes were sorted into potential themes, and all relevant coded data extracts were collated within the identified themes [13]. The themes were then reviewed for the coded extracts and the entire dataset. In the case of conflicts among researchers, the themes were refined, split, combined, or discarded. Each theme was refined in terms of scope and focus. Clear definitions and names were developed for each theme. At this stage, RO, AY, and CS discussed the content until a consensus was reached. RO recorded the findings using vivid and compelling examples to produce a report emphasizing the relationship between the analysis, research questions, and the literature. Data were managed using NVivo 11 (QSR International, Melbourne, Australia).

Reflexivity

This study was conducted collaboratively through interactions between the researchers and participants. The research team had diverse expertise and perspectives on rural community care. RO is a family physician and public health professional with a master's degree in public health and family medicine and has experience conducting qualitative and quantitative research on rural community healthcare and medical education. AY is a community nurse and a representative of a Community Nursing Company that has conducted qualitative research on rural community health care. CS is a medical educator and professor at a medical university who graduated from a medical university specializing in community healthcare management and education. To minimize bias, each idea related to community care was discussed by analyzing the research content of the individual data. Alternative viewpoints were explored in the data interpretation stage.

Ethical considerations

All participants were explained and consented to participate in this research before starting the education. The Unnan City Hospital Clinical Ethics Committee approved the study protocol (No. 20230008).

## Results

Results of the thematic analysis

**Figure 1:**
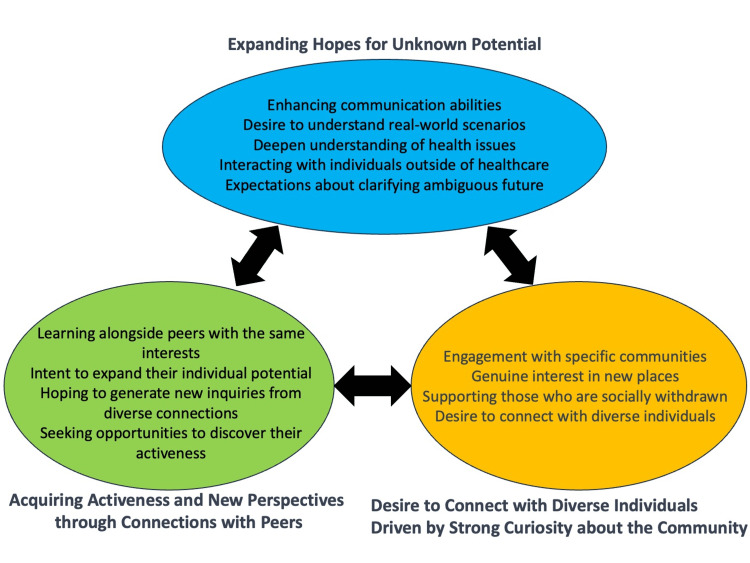
Thematic representation of healthcare students’ motivations in community organizing

Through a thematic analysis of the thoughts and motivations of healthcare profession students participating in community-organizing activities, three themes were developed: expanding hopes for unknown potential, acquiring activeness and new perspectives through connections with peers, and desire to connect with diverse individuals driven by a strong curiosity about the community (Table 1). The conceptual figure is presented in Figure 1.

**Table 1:** Results of the thematic analysis

Theme	Explanation
Expanding hopes for unknown potential	There is an increased interest in enhancing communication abilities. Among the students, there is a desire to understand real-world scenarios and deepen their understanding of health issues. They aim to gain new perspectives by stepping out of their usual interpersonal relationships and interacting with individuals outside of health care. They are motivated by techniques that engage with the community. There were expectations about how much they could change by participating. They desired to clarify ambiguous parts of their future through tangible experiences.
Acquiring activeness and new perspectives through connections with peers	Students were motivated by learning alongside peers who shared the same interests. There was intent to expand their individual potential. The aspiration to increase the number of peers with similar consciousness led to participation. They hoped to generate new inquiries from diverse connections. They were seeking opportunities to discover their own activeness.
Desire to connect with diverse individuals driven by a strong curiosity about the community	Students wanted to learn how to engage with specific communities through tangible experiences and desired to understand specifically how to be involved with a community. Participation was driven by a genuine interest in new places. They aspired to learn how they can engage in the activities of community members and influence their health status. There was increasing interest in supporting those who are socially withdrawn, as well as a desire to connect with diverse individuals in the community.

Expanding hopes for unknown potential

Participants had various thoughts about their professional careers. During formal high school and university education, students discuss their professional careers with colleagues from similar backgrounds. One participant stated, “I created only the same future with my peers in school” (Participant 3). Thus, there is increased interest among participants in enhancing personal communication abilities with various people outside of the fields in which they will work as future professionals. One participant stated, “I hope to have a dialogue with various students and citizens to broaden my perspectives” (Participant 6). By participating in the educational program, participants hoped to learn more about various specialties in addition to their own.

Participants wanted to understand real-world scenarios and deepen their understanding of health issues. Through their usual experiences, participants understood health issues from their own perspectives. Through this educational program, participants hoped to acquire perspectives that differed from those of professionals in their fields. One participant stated, “I hope to learn health issues from the different perspectives to take better care of patients in the future” (Participant 1).

Furthermore, participants aimed to gain new perspectives by stepping out of their usual interpersonal relationships and interacting with different worlds and individuals outside the healthcare setting. Participants were motivated to collaborate with people who were not healthcare professionals, with one explaining, “I would like to collaborate with people outside of my future professional peer group and learn completely different perspectives because I need to collaborate with various professionals in real life” (Participant 17.) They were also motivated to acquire new techniques to engage with the community. There were expectations regarding how much they could change citizens' motivation for health and health-seeking behaviors through their participation. They wanted to clarify the ambiguous aspects of their future through tangible experiences. One participant stated, “I hope to learn various communication tools with people outside of medicine. By learning with various students from different fields, I look forward to clarifying what I can do as a professional in the future” (Participant 2).

Acquiring activeness and new perspectives through connections with peers

Participants had various motivations for acquiring new knowledge about community organizations and health promotion. However, they did not have a broad range of colleagues at their universities to discuss their interests satisfactorily. One participant stated that “universities may not have many students who are motivated to learn about the community and work there. Students may not even be aware of this possibility” (Participant 20). By being aware of this education and being motivated to learn alongside peers who share the same interests, there was the intent to expand their potential. One participant explained, “Sharing my ideas can drive my understanding of community care and activities in communities. I will learn about the reality of community activities and skills” (Participant 24). Thus, participating in this educational program motivated them to learn new things based on their own interests.

The aspiration to increase the number of peers with a similar consciousness led to participation. Participants believed they needed more peers with the same motivations to learn about community organization and care. Through their participation, they hoped to meet peers and expand their relationships. One participant stated, “I hope to meet more peers and increase my relationship with people who are interested in community care. The expanded relationships motivated me to learn more” (Participant 26). In addition, participants hoped to generate new inquiries from diverse connections. Through diverse relationships, participants sought opportunities to discover new activities. One participant stated, “Diversity is essential, and I hope to learn from diverse students in the construction of learning relationships with them” (Participant 3).

Desire to connect with diverse individuals driven by a strong curiosity about the community

Participants wanted to learn how to engage with specific communities through tangible experiences. As the school curricula did not include learning content regarding community care and organizing, participants hoped to learn about community care and how to progress it with various professionals and citizens. One stated, “Learning about community care may not be possible in the present curricula. I searched for learning opportunities. This learning program was beneficial for me” (Participant 6). In addition, they wanted to understand how to become more involved in the community. Participants did not have any real experience in community care in their schools. They hoped to participate in community organizational activities and care. One explained, “I did not experience any community organizing and care activities. I am motivated to learn about them in real situations by collaborating with various people” (Participant 10).

Participation was also driven by genuine interest in new places. Participants usually learned only in school and reported not learning frequently outside this setting. Changing learning situations can motivate students to learn about their future specialties. One participant stated, “I am motivated to learn outside of the university. I can be refreshed by learning outside and acquiring new perspectives regarding my usual learning” (Participant 29). In addition, they aspired to learn how to engage in community activities and influence their health status. By participating in activities with community members, they hoped to understand the social issues affecting community health. There has been increasing interest in supporting socially withdrawn people on social issues, leading to a desire to connect with diverse individuals in the community. One participant stated, “I am interested in collaborating with community members and understanding the relationships between their social issues and health conditions. Social isolation and vulnerability in schools have also been discussed. I hope to learn more about these issues by participating in social activities to solve them” (Participant 8).

## Discussion

This study offers nuanced insights into the thoughts and motivations of healthcare professional students engaged in community-organizing education. Reflecting on an earlier article, the thematic analysis underscores several essential facets of students' aspirations and motivations.

One prominent theme was students' yearning to transcend traditional educational frameworks in favor of tangible experiences. This aligns with Kolb's experiential learning theory, which suggests that learning arises from transforming direct experience into knowledge [14]. Additionally, another study emphasizes that professional education should encompass "becoming a professional," which requires melding academic expertise with real-world complexities [15]. This improves students' grasp of theoretical concepts and shapes their professional identity [16]. Students' drive to navigate beyond their academic confines underscores their quest to become comprehensive and holistic professionals [17], which, in turn, highlights the need for curricula that offer more integrated experiential opportunities.

Community learning extends beyond understanding communities and underscores the importance of nurturing relationships within professional groups. This observation is consistent with Vygotsky's social development theory, which emphasizes the fundamental role of social interaction in cognitive development [18]. According to this theory, knowledge is co-constructed through interaction, making peer engagement a vital component of the learning process. In community care, students recognize the value of collaborative learning [19]. Indeed, they intuitively understand that their professional journey is deepened by their individual experiences, collective reflections, shared challenges, and mutual aspirations [20]. This insight reinforces the importance of fostering collaborative learning environments where students can actively engage with their peers, promoting more prosperous and diverse learning outcomes.

Students' intrinsic motivation to delve deeply into community dynamics highlights the importance of authentic engagement in theoretical knowledge. This mirrors previous studies' arguments on experiential learning, suggesting that genuine experiences foster more profound understanding and critical thinking [21]. Moreover, this curiosity reveals students' recognition of the interconnectedness of health, society, and the environment, as emphasized in broader discussions on the social determinants of health [22]. Given that health is not merely an individual's concern but is interwoven with the community fabric, students' keenness to engage directly with diverse community members signifies a shift toward a more holistic and integrative view of health and well-being [23]. This perspective, in sync with the extant literature, highlights the need to prioritize community immersion in health-related curricula.

This study has several limitations. First, it relied primarily on self-reported data, which may be susceptible to social desirability bias if participants responded in a manner that they perceived as favorable. This could have skewed the authenticity of the responses. Second, the participant pool was limited to a single educational setting with limited participants, which may not have captured diverse student experiences across different institutions or regions. Therefore, the findings may not be generalizable to all healthcare students. Third, although rigorous, thematic analysis is inherently interpretative and may be influenced by researchers' biases and preconceptions. This study did not examine the long-term effects or implications of students' experiences but focused more on immediate reactions and perceptions. Future research should address these limitations to provide a more comprehensive understanding of the topic.

## Conclusions

The thematic analysis affirmed the vital role of community-organizing education in shaping future healthcare professionals. The students' reflections here corroborate the larger narrative in the academic literature concerning the importance of field experiences, peer interactions, and community engagement. Thus, there is a strong need for curriculum reform to integrate such modules comprehensively, ensuring that healthcare students are equipped with sufficient medical knowledge and a deeper understanding of community dynamics and the social aspects of health. These findings suggest a potential avenue for further research to understand how community engagement influences students' long-term career trajectories and community health outcomes.
